# Low-Cost Potentiometric Sensor for Chloride Measurement in Continuous Industrial Process Control

**DOI:** 10.3390/molecules27103087

**Published:** 2022-05-11

**Authors:** Martina Vizza, Patrick Marcantelli, Claudia Giovani, Walter Giurlani, Paolo Giusti, Claudio Fontanesi, Massimo Innocenti

**Affiliations:** 1 Department of Chemistry “Ugo Schiff”, University of Florence, Via della Lastruccia 3, 50019 Sesto Fiorentino, FI, Italy; patrick.marcantelli@unifi.it (P.M.); claudia.giovani@unifi.it (C.G.); walter.giurlani@unifi.it (W.G.); 2 National Interuniversity Consortium of Materials Science and Technology (INSTM), Via G. Giusti 9, 50121 Firenze, FI, Italy; 3 CDR S.R.L., Via degli Artigiani 6, 50055 Ginestra Fiorentina, FI, Italy; paolo@mediared.it; 4 Department of Engineering “Enzo Ferrari” (DIEF), University of Modena, Via Vivarelli 10, 41125 Modena, MO, Italy; claudio.fontanesi@unimore.it; 5 Institute of Chemistry of Organometallic Compounds (ICCOM), National Research Council (CNR), Via Madonna del Piano 10, 50019 Sesto Fiorentino, FI, Italy; 6 Center for Colloid and Surface Science (CSGI), Via della Lastruccia 3, 50019 Sesto Fiorentino, FI, Italy

**Keywords:** ISE sensor, chloride determination, OCP, potentiometry, industrial process control, low-cost sensor, continuous monitoring, in situ measurements

## Abstract

Recently, the new updates in legislation about drinking water control and human health have increased the demand for novel electrochemical low-cost sensors, such as potentiometric ones. Nowadays, the determination of chloride ion in aqueous solutions has attracted great attention in several fields, from industrial processes to drinking water control. Indeed, chloride plays a crucial role in corrosion, also influencing the final taste of beverages, especially coffee. The main goal is to obtain devices suitable for continuous and real-time analysis. For these reasons, we investigated the possibility to develop an easy, low-cost potentiometric chloride sensor, able to perform analysis in aqueous mediums for long immersion time and reducing the need of periodic calibration. We realized a chloride ion selective electrode made of Ag/AgCl sintered pellet and we tested its response in model solutions compatible with drinking water. The sensor was able to produce a stable, reproducible, and accurate quantification of chloride in 900 s, without the need for a preliminary calibration test. This opens the route to potential applications of this sensor in continuous, in situ, and real time measurement of chloride ions in industrial processes, with a reduced need for periodic maintenance.

## 1. Introduction

In the last few decades, the continuous updates in legislation concerning human health, wastewater, and drinking water control have contributed to the necessity of developing new chemical low-cost sensors [1]. In particular, devices suitable for continuous, real-time, and online analysis would be preferable for the monitoring of different parameters [2,3,4,5]. Among these, the detection and quantification of chloride ion concentration in aqueous conditions is of growing concern in different fields, spanning from industrial process control to food, beverage, and soil quality [6]. For example, in metal pipes, the presence of chloride reacting with metal ions can determine the formation of soluble salts, potentially increasing the level of metals in drinking water. Besides that, the chloride content can influence the organoleptic properties of drinking water, and this is crucial for the final taste of beverages such as coffee [7]. Nowadays, the greatest concern about chloride monitoring is related to the fact that this anion plays an important role in corrosion processes (e.g., formation of oxide films on copper-based alloys in marine environment, crevice and concrete corrosion) [8,9,10]. For example, chloride can contribute to the enhancement of galvanic corrosion in lead and metal pipes [11]. For these reasons, the control of parameters affecting corrosion, including chloride concentration, could allow the early detection of future problems and the planning of corrective actions to ensure the suitability and durability of devices, systems, and infrastructures [10]. Nowadays, the quantification of chloride ions can be performed with many different devices, such as optical fibre sensors [12,13], fibre grafting sensors [14,15], and electrochemical sensors [6]. The latter ones are of particular interest in analytical research for many different applications, including not only the realization of wearable health devices [16,17,18,19,20], but also soil and water quality control [6]. The electrochemical sensors of longest history and largest number of applications are ion-selective electrodes (ISEs), which allow fast and maintenance free analysis with usually long lifetime and durability [21]. For many years, sensor research has focused on the development of rugged and low-cost commercial devices, which could be mass produced by the industry. One of the main issues to be considered when implementing a technological innovation in practice is the total cost per analysis [21]. This topic is nowadays more and more addressed, but what is high or low cost basically depends on the specific final application of the sensor. For example, a high-density sensor network is usually demanded in environmental and agricultural monitoring and the overall cost of the investment has to be carefully pondered, especially taking in account the average sensor lifetime [1]. Besides that, the capital related to consumables, service, and labour required by the commercial manufacture is another relevant factor in the design of a commercial ISE [21]. The literature reports a great variety of chloride ion selective sensors, with different detection principles [22]. ISEs based on polymeric membranes are of particular interest, since they present potential applications for clinical, environmental, and industrial purposes [23,24,25]. However, the lifetime of these devices is still debated [26,27]. Furthermore, the selectivity of such chloride sensors is based on ionophore ion carriers, which can be carcinogenic, with low environmental compatibility [28,29,30,31,32]. All-solid-state miniature planar selective chloride electrodes can be prepared by using a screen-printing technology, still presenting only two months lifetime [33]. As a consequence, one of the easiest, most common, and environmentally friendly ISE chloride sensors is made of Ag/AgCl [10]. This type of electrode is stable and can be easily prepared, also showing a good sensitivity and durability with a Nernstian response to the variations of chloride activity [10,22]. In general, Ag/AgCl ISEs are used in potentiometric sensors, which are characterized by small dimensions and low energy consumption. Therefore, they are perfectly suitable for clinical and environmental analysis, as well as process control [21]. The sensing mechanism of a potentiometric sensor is based on the use of a potentiometer, which is used to register the open-circuit potential (OCP) response of the Ag/AgCl ISE versus a known reference electrode (RE), in the absence of a current flow [1,6]. For an Ag/AgCl sensor, the OCP shows the activity of chloride ions in the medium, following the Nernst equation [34,35] (Equation (1)).

(1) $$E_{Ag/AgCl\;}=E_{Ag/AgCl}^{0}-2.303\frac{RT}{nF}lg\left[a_{Cl^{-}}\right]\;$$

where $E_{Ag/AgCl\;}$ is the measured OCP of the sensor, $E_{Ag/AgCl\;}^{0}$ is the standard electrode potential for the Ag/AgCl electrode (V), $a_{Cl^{-}}$ is the activity of the chloride ions (mol·dm^−3^) near the elctrode, R is the gas constant (J·mol^−1^·K^−1^), F is the Faraday constant (C·mol^−1^), and T is the absolute temperature (K). According to Equation (1), there is a linear relationship between the OCP of the sensor and the logarithm of the chloride ions activity in solution. Therefore, it can be said that the OCP response of the sensor reflects the activity of chloride at the Ag/AgCl interface and, consequently, the chloride ions concentration in the medium. The chloride concentration in the medium can be derived from the sensing anion activity using the activity coefficient (c). In this way, the OCP signal can be related to logarithm of the ionic chloride concentration in solution, allowing the quantification of Cl^-^ in a certain linear range [35,36].

The performance of a chloride sensor is determined not only by the efficiency of the ISE, but also by the stability of the reference electrode [1,6]. Mostly, electrodes of second kind are used as REs due to their reliability and durability, including the saturated Ag/AgCl and calomel electrodes. The latter ones are preferably avoided, due to the growing attention towards environmental sustainability. Therefore, the saturated Ag/AgCl RE can be defined as the easiest, most common, and widespread reference electrode for chloride sensors [1]. In the last few decades, several attempts have been made to produce innovative Ag/AgCl REs, which could be miniaturized avoiding the disadvantage of internal liquid or gel fillings. Sophocleus and Atkinson reported the possibility to use small dimension screen-printed Ag/AgCl REs for potentiometric applications, but those reference electrodes never reached the final commercialization stage because of their controverted reliability and durability [1,6]. The literature also reports several reviews on innovative all-solid-state REs with solidified reference electrolyte [37], which could overcome the disadvantages of liquid components in the miniaturization process. However, to date no type of solid-state reference electrode presents low-cost, commercial production feasibility, stability, and lifetime fully comparable to conventional saturated Ag/AgCl REs [1,38]. The latter ones remain the most reliable reference electrodes to be used in potentiometric chloride sensors [1]. Another issue when dealing with ISEs is the fact that the response of the sensor can sometimes deviate from the ideal Nernstian slope [39,40,41,42,43]. Besides that, ISEs can be subjected to drift in their response. This can be accommodated through calibration of the sensor with standards of changing ionic concentrations, so that the calibrated slope can be used in place of the ideal slope to calculate the chloride concentrations in unknown samples [20]. However, the need for periodic calibrations requires time-consuming maintenance and intervention. These additional factors can significantly increase the total cost of the analysis, representing a significant disadvantage for continuous and real time measurements.

For these reasons, we focused on the development of an easy and low-cost potentiometric chloride sensor, able to perform reliable analysis in aqueous medium for a long immersion time, reducing the need of periodic calibrations. We realized a chloride ISE made of Ag/AgCl sintered pellet and we measured the OCP versus a saturated Ag/AgCl RE in 10 °fH–40 °fH model solutions, containing 5–100 mg/L of chloride. In this way, aqueous medium with similarities to drinking water for food and beverage industry was simulated. The stability and reproducibility of the sensor were tested in relation to the background conductivity of the different model solutions. The possibility to reduce the need for periodic calibration was investigated, in order to expand the possible applications of low-cost chloride determination in continuous, in situ, and real-time measurements.

## 2. Materials and Methods

We used NaCl, MgSO_4_·7H_2_O, and CaSO_4_·2H_2_O salts to prepare solutions with different chloride content (5 mg/L, 10 mg/L, 20 mg/L, 30 mg/L, 40 mg/L, 50 mg/L, 75 mg/L, 100 mg/L) and water hardness levels (10 °fH, 20 °fH, 30 °fH, 40 °fH), with MilliQ water (18 MW, Merk Millipore, Burlington, MA, USA). The working ISE electrode was made of a commercially available 2 mm × 4 mm Ag/AgCl sintered pellet. We measured the OCP signal between the working and the reference electrode (saturated Ag/AgCl) using the VC8145 dual display digital multimeter (Shenzhen Vicimeter Technology Co., Shenzhen, China). Each OCP measurement was performed in stirring conditions during the recording time of 900 s. The temperature of the solutions was kept between 20 °C and 25 °C. The OCP measurements were performed at room temperature and pressure.

## 3. Results

The sensor’s response was studied in solutions with different chloride content and water hardness. The linearity of the OCP signal was evaluated in each model solution. The reproducibility of the sensor was studied by means of statistical analysis and the possibility to reduce the need for periodic calibration was investigated.

### 3.1. Study of the Sensor’s OCP Response in Solutions with Different Chloride Content and Water Hardness

**Figure 1:**
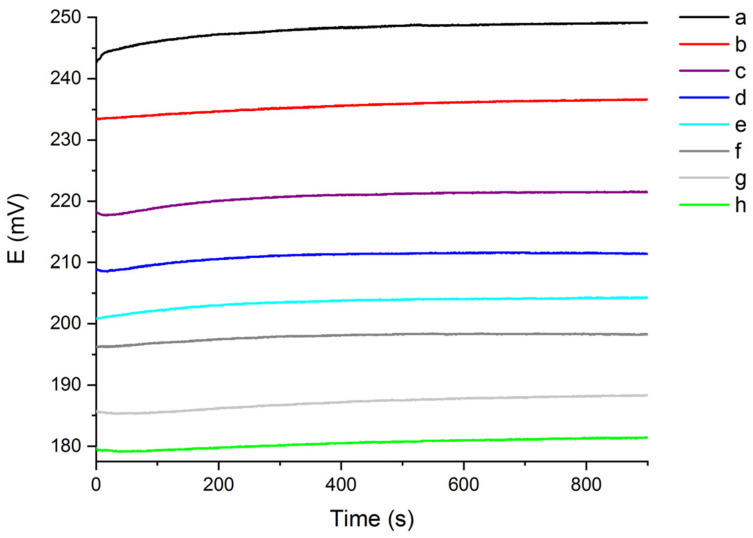
OCP signal recorded for 900 s in 10 °fH solutions, with different chloride content (a: 5 mg/L; b: 10 mg/L; c: 20 mg/L; d: 30 mg/L; e: 40 mg/L; f: 50 mg/L; g: 75 mg/L; h: 100 mg/L).

The chloride ion response of the testing sensor was studied by recording the OCP signal of the Ag/AgCl working electrode versus the saturated Ag/AgCl RE in stirred chloride aqueous solutions, characterized by different hardness levels (10–40 °fH) and chloride content (5–100 mg/L). The sensing test was firstly performed in the softer water solutions (10 °fH), whose hardness level was maintained constant while varying the chloride content. Consequently, the only contribute to the changing of the aqueous medium’s conductivity was the sensing anion concentration. This was meant to preliminarily focus on the stability of the OCP as a function of the chloride content, avoiding any cross effect caused by variations of the background model solution’s conductivity. In each test, the OCP recording started in the aqueous medium with the lower chloride content (5 mg/L), which was gradually increased until reaching the highest value of 100 mg/L, as reported in Figure 1.

As shown in Figure 1, the OCP signal is inversely proportional to the chloride concentration in solution, so that the OCP increases while decreasing the chloride content. Furthermore, the OCP signal starts at lower values and then gradually increases during the recording time of 900 s, finally reaching a stable plateau. For each of the eight solutions analysed, the chloride sensor was able to produce a different OCP output signal as a function of the chloride content. The reversibility and sensitivity of the sensor in 10 °fH aqueous medium were evaluated by registering the OCP response in solutions with significantly different chloride content. In particular, by sharpening the decreasing concentration of chloride from 100 mg/L to 5 mg/L, in 900 s the OCP signal returned approximately to the initial and expected response of 248 mV. This demonstrates the good sensitivity and reversibility of the sensor in 10 °fH solutions in the entire chloride content range examined [35].

**Figure 2:**
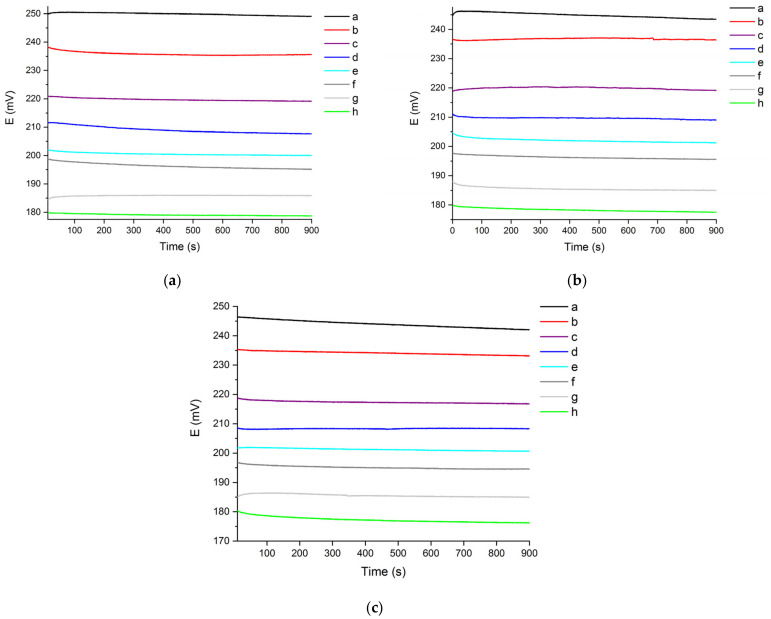
Chloride sensor’s OCP signal recorded in 900 s, in (**a**) 20 °fH, (**b**) 30 °fH and (**c**) 40 °fH solutions with different chloride content (a: 5 mg/L; b: 10 mg/L; c: 20 mg/L; d: 30 mg/L; e: 40 mg/L; f: 50 mg/L; g: 75 mg/L; h: 100 mg/L).

The effect of the solution’s background conductivity on the stability of the response was studied by recording the OCP signal in chloride solutions with hardness levels between 20 °fH and 40 °fH (see Figure 2). In analogy with the test of the OCP signal in 10 °fH solutions (see Figure 1), the water hardness was maintained fixed, while gradually increasing the chloride concentration from 5 mg/L to 100 mg/L.

The curves shown in Figure 2 are flatter than those recorded in 10 °fH solutions (see Figure 1). This suggests that, during the 900 s recording time, the sensor’s response in 20–40 °fH aqueous mediums is subjected to smaller variations than the ones recorded in 10 °fH solutions. The curves in Figure 2 reach stable plateau values in of 900 s, similarly to the OCP’s behaviour in 10 °fH solutions (see Figure 1). As reported in Figure 2, the sensor is able to produce distinct OCP output signals depending on the chloride content in 20–40 °fH model solutions, showing good reversibility after drastic concentration variations. This suggests that, for a certain chloride content, the stability and reversibility of the OCP signal produced by the sensor are not significantly affected by water hardness variations. This evidence is particularly important, suggesting the chloride sensor’s ability to produce an OCP signal that is not affected by variation of the solution’s hardness levels and, therefore, of the solution’s background conductivity.

### 3.2. OCP Signal’s Linearity in Solutions with Different Hardness Levels and Chloride Content

**Figure 3:**
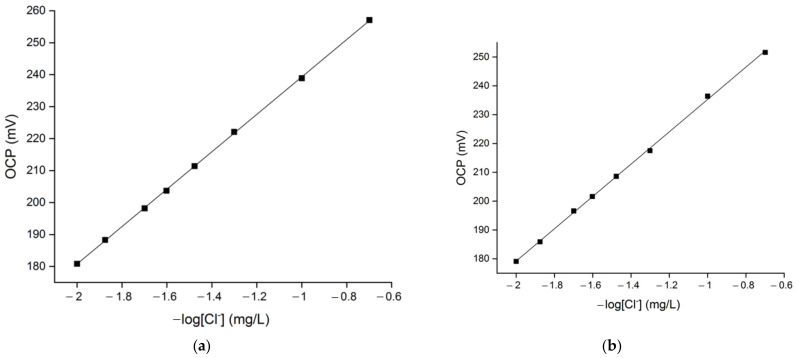

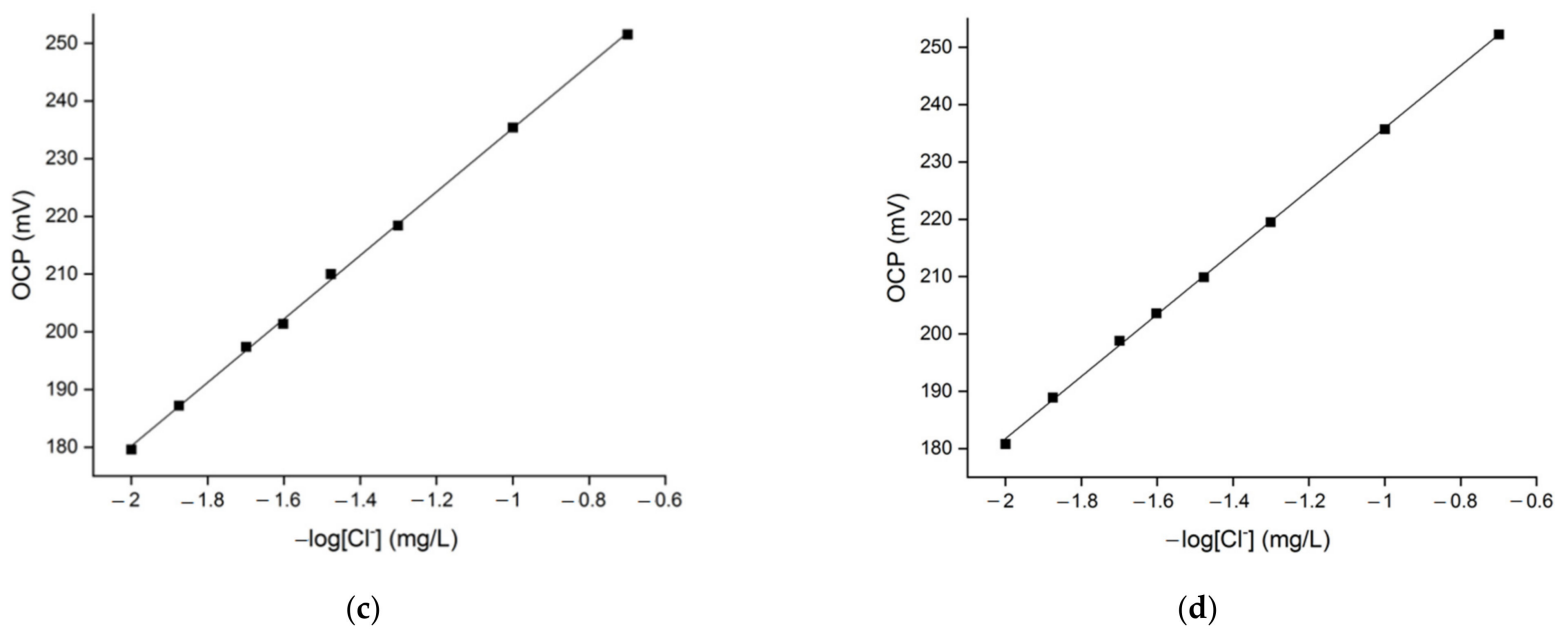
Chloride sensor’s linearity response in (**a**) 10 °fH, (**b**) 20 °fH, (**c**) 30 °fH, (**d**) 40 °fH solutions with different chloride content (5–100 mg/L).

Figure 3 shows the sensor’s chloride response in solutions with fixed water hardness in the range between 10 °fH and 40 °fH, as a function of the chloride concentration. The coefficient of determination was derived by means of linear regression. The sensor responded with high accuracy to the entire chloride concentration in the model solutions examined. Indeed, the coefficient of determination values (R^2^) shown in Table 1 are higher than 0.999. The slope of the calibration curves varies from 58.5 mV to 54.2 mV and decreases with the increase of the solution’s hardness value (see Figure 3). The calibration slope curve presents an approximately Nernstian value (58.5 mV) in 10 °fH chloride solutions, while a slight deviation from this theoretical behaviour can be observed for the sensor’s response in harder water mediums (20–40 °fH). This is due to the fact that the theoretical Nernstian slope is derived from the assumption of thermodynamic equilibrium and spontaneity of the reactions taking place at the sensor’s surface in standard conditions, while the experimental Nernstian slope depends on the specific conditions of the experiment. Variations of the solution conductivity due to the different water hardness values can produce calibration curves with slopes that assume different values and derive from the predicted Nernstian behaviour. However, the calibration curves reported in Figure 3 are characterized by slope values that are fully acceptable, indicating a good sensitivity of the sensor [3,28,35].

### 3.3. Reproducibility of the Chloride Sensor’s OCP Response

The response of the sensor was studied in terms of reproducibility by repeatedly recording the OCP signals in stirred solutions with different hardness and chloride concentrations. In each measurement set, the hardness level was maintained constant in the range between 10 °fH and 40 °fH, while the chloride content was gradually increased from 5 mg/L to 100 mg/L. OCP signals were recorded after 15 min for each chloride content and hardness value in solution. The measurements were repeated 10 times for each combination of chloride concentration and water hardness. As a consequence, 320 OCP sensors’ responses were collected and statistically analysed, calculating their arithmetical average values and standard deviation (see Table 2). The highest standard deviation reported in Table 2 is relative to the sensor’s OCP response in 10 °fH solutions containing 5 mg/L of chloride. In the other cases, the standard deviation is characterized by values between ±1 mV and ±3 mV, except for the ±3.7 standard deviation associated to the sensor’s OCP response in 20 °fH solutions with 5 mg/L chloride content. Therefore, it can be said that the sensor is able to produce a reproducible OCP signal in 10 °fH solutions, with a chloride content between 10 mg/L and 100 mg/L, as well as in 20–40 °fH solutions containing 5–100 mg/L of chloride. Based on our measurements, the limit of detection (LOD) of the sensor is 10 mg/L in 10 °fH solutions, while it is 5 mg/L in 20–40 °fH solutions in our examined experimental conditions, that is without a preliminary calibration curve.

Then, the possibility to apply the sensor to chloride quantification, drastically reducing the need of periodic calibration, was investigated. For a certain chloride concentration, we calculated the arithmetical average and standard deviation of the OCP signals produced by the sensor in solutions with different water hardness levels (see Table 2). Those results are reported in Table 3.

Considering the fully acceptable standard deviations in Table 2; Table 3, the sensor is able to produce an esteem of the chloride content also without a preliminary calibration curve. Indeed, once the sensor’s OCP response has been experimentally recorded, this value can be compared with the arithmetical averages reported in Table 3. In such way, it is possible to determine an average chloride’s concentration in the examined range, independently of the background conductivity determined by the different water hardness levels. This represents a significant advantage for a chloride sensor to be potentially used for continuous, online, and real-time monitoring of drinking water.

**Figure 4:**
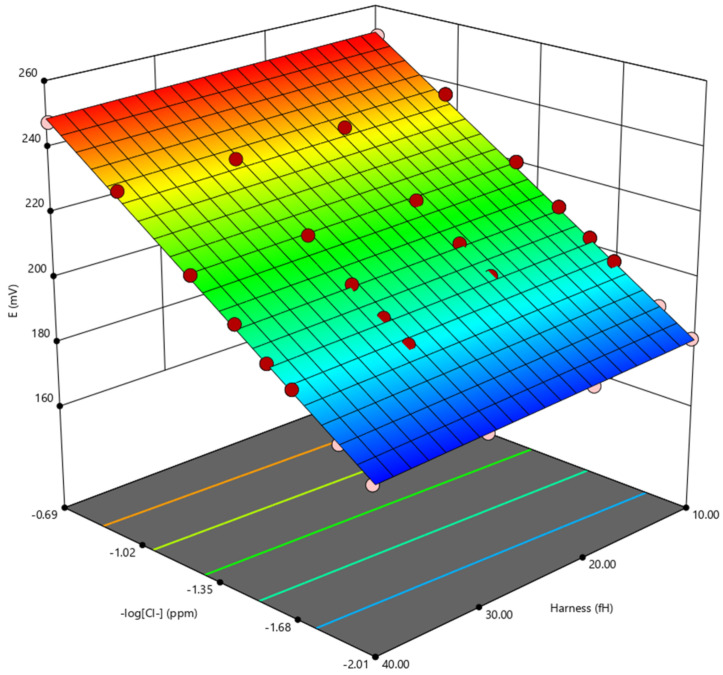
3D plot obtained using the multivariate analysis.

The measured potential recorded with various amounts of chlorides and hardness reported in Table 2 were analysed with a multivariate analysis approach to extrapolate the relation between the negative logarithm of the chloride content (–log[Cl^–^]), hardness (fH), and potential (E). The data were fitted with a cubic function (Equation (2) and Figure 4).

E = 2.885 × 10^2^ + 5.269 × 10 × (−log[Cl^−^]) − 5.123 × 10^−2^ × fH + 4.489 × 10^−2^ × (−log[Cl^−^]) × fH (2)

## 4. Conclusions

In this work, we focused on the development of a low-cost potentiometric chloride sensor, which could be easily fabricated and able to perform reliable analysis in aqueous medium for a long immersion time. This was mainly aimed at reducing the need for periodic calibrations and, consequently, the total cost per analysis. We used a chloride ISE made of Ag/AgCl sintered pellet and we measured the OCP at room temperature versus a saturated Ag/AgCl RE in stirred 10 °fH–40 °fH aqueous solutions, containing 5–100 mg/L of chloride. In this way, an aqueous medium with similarities to drinking water for the food and beverage industry was simulated. We observed that the OCP signal produced by the sensor was inversely proportional to the chloride concentration in solution, so that the OCP increased while decreasing the chloride content. Furthermore, the OCP signal reached a stable plateau value after 900 s. This demonstrated the good sensitivity and reversibility of the sensor in the examined experimental conditions. The sensor was also able to produce distinct OCP output signals depending on the chloride content in 10–40 °fH model solutions, showing good reversibility after drastic concentration variations. Therefore, we can say that the stability and reversibility of the sensor’s response were not significantly affected by water hardness variations. This evidence is of particular interest, showing the ability of the chloride sensor to produce an OCP signal not consistently affected by variations of the solution’s background conductivity. The sensor responded with high accuracy to the entire chloride concentration in the model solutions examined, with coefficient of determination values (R^2^) higher than 0.999.

The response of the sensor was then studied in terms of reproducibility for each combination of chloride concentration and water hardness. Hence, 320 OCP sensors’ responses were collected and statistically analysed. The standard deviation values showed the ability of the sensor to produce reproducible OCP signals in the investigated range of chloride and water hardness.

In order to reduce the total cost per analysis and maintenance of the sensor, we investigated the possibility to apply the sensor to chloride quantification, drastically reducing the need for periodic calibration. We found that the reproducibility of the sensor was not significantly affected by variations of the background conductivity in solution, caused by the changing of the hardness levels in the examined range. Furthermore, the sensor was able to produce an estimate of the chloride content without the need for a preliminary calibration curve. The possibility to obtain reliable quantifications of the chloride content in solution without the need for high-cost maintenance and periodic calibrations presents several advantages for potential applications in industrial process control, such as corrosion, as well as in the analysis of water destined for the production of food and beverages. In addition, a future perspective in the use of the developed sensor could be its applicability in extremely salty media (0.1–1 mol/L). This could be of great importance in the industrial field, for several applications. Therefore, the developed potentiometric and low-cost chloride sensor could potentially be applied in continuous and in situ monitoring of the chloride content in several different contests, ranging from industrial to drinking water.

## Figures and Tables

**Table 1:** Slope, intercept and coefficient determination values of the sensor’s calibration curves in solutions with different hardness and chloride content (see Figure 3).

Water Hardness (°fH)	Slope	Intercept	Coefficient of Determination (R^2^)
10	58.5	297.8	0.9998
20	56.1	291.3	0.9992
30	55.1	290.3	0.9993
40	54.2	290.1	0.9995

**Table 2:** Arithmetical average and standard deviation of the sensor’s OCP response in 10–40 °fH solutions with different chloride content (5–100 mg/L).

Water Hardness (°fH)	Chloride Content (mg/L)	OCP Arithmetical Average (mV)	Standard Deviation
10	5	250.2	6.4
	10	236.5	2.0
	20	220.7	1.6
	30	210.1	1.4
	40	203.1	1.1
	50	197.9	0.9
	75	188.1	1.3
	100	181.1	1.0
20	5	247.2	2.6
	10	234.6	1.8
	20	218.2	1.4
	30	208.9	1.4
	40	201.9	1.2
	50	196.2	1.6
	75	186.2	1.6
	100	178.8	1.6
30	5	244.9	3.7
	10	234.0	1.8
	20	217.7	1.9
	30	207.3	2.7
	40	200.6	2.2
	50	195.3	2.6
	75	184.3	2.3
	100	177.7	1.9
40	5	247.9	2.2
	10	234.0	1.3
	20	216.7	1.7
	30	207.1	1.2
	40	199.2	1.5
	50	194.5	1.3
	75	184.1	1.1
	100	176.5	1.1

**Table 3:** Arithmetical average and standard deviation values of the OCP response recorded for a certain chloride concentration (5–100 mg/L) in solutions with different hardness levels (10° fH–40 °fH) (see Table 2).

Chloride Content (mg/L)	Arithmetical Average of OCP Recorded in 10 °fH–40 °fH Solutions	Standard Deviation
5	247.5	3.9
10	234.8	1.9
20	218.3	2.1
30	208.3	2.2
40	201.2	1.9
50	196.0	2.1
75	185.7	2.3
100	178.5	2.0

## Data Availability

Not applicable.
